# Perirenal abscess as a cause of intrauterine fetal death: A case report

**DOI:** 10.1016/j.eucr.2023.102363

**Published:** 2023-02-27

**Authors:** Maher Al-Hajjaj, Muna Alqralleh, Asmaa Omar, Mohamed Abdelmalik, Nora Mohammed

**Affiliations:** a Department of Urology, University of Aleppo, Aleppo, Syria; b Department of Obstetrics & Gynecology, Jordanian Royal Medical Services, Amman, Jordan; c Faculty of Medicine, Mansoura University, Dakahlia, Egypt; d Alzaim Alazhari University, Khartoum, Sudan; e University of Dongola, Khartoum, Sudan

## Abstract

Urinary tract infection is one of the most common causes of hospital admission during pregnancy. We present a 27 years old woman who was at 28 weeks of pregnancy and she urinary tract infection developed to acute pyelonephritis. This condition developed to form perirenal abscess and intrauterine fetus death. Computed tomography scan showed right perirenal abscess. Ultrasonography is the imaging modality of choice during pregnancy because of its lack of ionizing radiation. Our patient developed perirenal abscess as a complication of untreated acute pyelonephritis. Pyelonephritis during pregnancy is considered a serious condition and should be treated promptly.

## Highlights

• Acute pyelonephritis is the most common bacterial infection during pregnancy.
• Untreated pyelonephritis will lead to serious complications on the patient and the fetus.
• Perirenal abscess may develop as a complication of pyelonephritis.
• Intrauterine fetal death is a rare complication of perirenal abscess during pregnancy.

## 1 Introduction

Acute pyelonephritis complicates about 1–2% of pregnancies and is one of the leading causes of obstetric hospitalization. It is most frequently seen in the second trimester and usually affects the right kidney.^1^

The diagnosis of renal infection is based mainly on the clinical findings of fever, chills and flank pain. Ultrasonography is the imaging modality of choice during pregnancy because of its lack of ionizing radiation. In non-gravid patients, contrast-enhanced CT is the modality of choice to detect acute pyelonephritis as well as renal abscess.^2^

It is a serious condition that can have severe maternal and fetal complications.

We had a rare case of acute pyelonephritis in a pregnant woman which led to fetal death.

## 2 Case presentation

A 27-year-old female patient presented to emergency department with three days of fever, chills, and right lower back pain. She was pregnant at 28 weeks. Past medical history was unremarkable. Vital signs were as follow: blood pressure was 100/60 mmHg, pulse was 86/min, spo2 was 91%, and respiratory rate was 22/min. Physical examination revealed right flank pain. The gynecological examination showed slight intravaginal blood and fetal bradychardia. Laboratories are shown in Table 1.

**Table 1:** Laboratory findings.

Wight blood cell count	Hemoglobine	Platelets	Creatinine	CRP	Urea	Glucose	Na+	K+
17 × 10^5^/ml	10.5 × 10^5^gr/dl	300 × 10^5^/mcl	1.1 mg/dl	89	41 mg/dl	85 mg/dl	142 mEq/L	4.3 mEq/L

Abdominal and pelvis ultrasound showed right renal hydronephrosis grade one and a heterogenic appearance of the perirenal fat. In addition, there was an alive fetus intrauterine. We started with third generation of cephalosporin. The patient refused to insert a double j ureteral stent. Urine culture was negative.

**Fig. 1:**
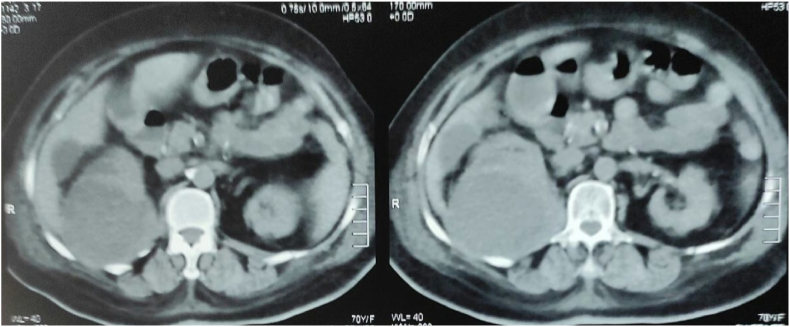
Computed tomography showes right perirenal abscess.

Evaluation of the patient after 48 hours demonstrated continuous fever 39.5C severe pelvic pain. A repeated ultrasound showed fetal death in utero. As a result, the patient underwent an induction of labor. Later, the patient had a continuous fever. We performed computed tomography (CT) scan of abdomen and pelvis on the fourth day of admission which showed right perirenal abscess (Fig. 1). We performed right renal abscess drainage and pus showed the presences of Escherichia coli.

After 15 days of drainage, we discharged the patient in a good state. Follow-up for one month showed a complete resolution of the abscess.

## 3 Discussion

Urinary tract infections are the most common bacterial infections during pregnancy. This is due to the anatomical and physiological changes in the urinary tract in pregnant women.^1^

The incidence is approximately 2.5%, which accounts for approximately 14 per 1000 deliveries or 53 per every 10,000 deliveries. This is almost a ten-fold increase compared to nonpregnant women, in whom the incidence is approximately 15–17 per 10,000.^3^

During pregnancy, asymptomatic bacteriuria occurs in 3% of patients and may be complicated by pyelonephritis with the risk of serious maternal and fetal complications. It is more frequent in the last weeks of pregnancy.^4^

The diagnosis of renal infection is based mainly on the clinical findings of fever, chills and flank pain^2^.

All suspected cases of pyelonephritis are hospitalized and treated with intravenous antibiotics until afebrile for 24 hours and symptomatically improved.^5^

We had a rare case of a pregnant woman who presented with symptoms of right acute pyelonephritis. Ultrasound showed 28 weeks intrauterine fetus. After the starting of intravenous antibiotics, the patient complained of persistent fever and severe pelvic pain. Ultrasound confirmed the death of the fetus. Induction of labor was started after taking the patient consent.

After that, the patient underwent abdominal and pelvis CT scan which showed right perirenal abscess.

Drainage of the abscess was the definite treatment. The culture of pus showed E. coli infection. Treatment with intravenous antibiotics for two weeks showed a full recovery.

## 4 Conclusion

Perirenal abscess is a rare cause of intrauterine fetus death. Pregnant women who have bactiuria should be treated seriously to avoid developing pyelonephritis.

## Sources of funding

This research did not receive any specific grant from funding agencies in the public, commercial, or not-for-profit sectors.

## Consent

Written informed consent was obtained from the patient's parents for publication of this case report and accompanying images, in line with local ethical approval requirements. No other requirements were stipulated.

## Declaration of competing interest

There was no conflict of interest.
